# Genotyping of methicillin-resistant *Staphylococcus aureus* in the Sultan Qaboos University Hospital, Oman reveals the dominance of Panton–Valentine leucocidin-negative ST6-IV/t304 clone

**DOI:** 10.1002/nmi2.47

**Published:** 2014-05-27

**Authors:** E E Udo, B A-H Al-Lawati, Z Al-Muharmi, S S Thukral

**Affiliations:** 1 Department of Microbiology, Faculty of Medicine, Kuwait UniversitySafat, Kuwait; 2 Department of Microbiology and Immunology, Faculty of Medicine and Health Sciences, Sultan Qaboos UniversityMuscat, Oman

**Keywords:** Antibiotic resistance, multilocus sequence typing, molecular typing, methicillin-resistant *Staphylococcus aureus*, Spa typing

## Abstract

The objective of this study was to determine the prevalence and distribution of methicillin-resistant *Staphylococcus aureus (*MRSA) genotypes circulating at a tertiary hospital in the Sultanate of Oman. A total of 79 MRSA isolates were obtained from different clinical samples and investigated using antibiogram, pulsed-field gel electrophoresis (PFGE), staphylococcal chromosome cassette mec (SCC*mec*), Spa typing and multilocus sequence typing (MLST). The isolates were susceptible to linezolid, vancomycin, teicoplanin, tigecycline and mupirocin but were resistant to tetracycline (30.4%), erythromycin (26.6%), clindamycin (24.1%), trimethoprim (19.0%), ciprofloxacin (17.7%), fusidic acid (15.2%) and gentamicin (12.7%). Molecular typing revealed 19 PFGE patterns, 26 Spa types and 21 sequence types. SCC*mec*-IV (86.0%) was the dominant SCC*mec* type, followed by SCC*mec*-V (10.1%). SCC*mec*-III (2.5%) and SCC*mec*-II (1.3%) were less common. ST6-IV/t304 (*n* = 30) and ST1295-IV/t690 (*n* = 12) were the dominant genotypes followed by ST772-V/t657 (*n* = 5), ST30-IV/t019/t021 (*n* = 5), ST22-IV/t852 (*n* = 4), ST80-IV/t044 (*n* = 3) and 18 single genotypes that were isolated sporadically. On the basis of SCC*mec* typing and MLST, 91.2% of the isolates were classified as community-associated MRSA and 8.8% of the isolates (consisting of four ST22-IV/t852, one ST239-III/t632, one ST5-III/t311 and one ST5-II/t003) were classified as healthcare-associated MRSA. The study has revealed the dominance of a Panton–Valentine leucocidin-negative ST6-IV/t304 clone and provided insights into the distribution of antibiotic resistance in MRSA at the tertiary hospital in Oman. It also highlights the importance of surveillance in detecting the emergence of new MRSA clones in a healthcare facility.

## Introduction

The burden of infections caused by methicillin-resistant *Staphylococcus aureus* (MRSA) is increasing among different patient populations globally 1–3. Following its initial report in 1961 4, MRSA has remained an important cause of infections in healthcare facilities and in the community globally 1–3. Although previously restricted to healthcare facilities, especially large tertiary-care facilities 5, MRSA has been increasingly identified as a major cause of community-associated infections in previously healthy hosts since the late 1990s 6–8. These new MRSA strains have been described as community-acquired or community-originated MRSA. Community-acquired MRSA can be distinguished from healthcare-associated MRSA isolates on the basis of patient risk factors such as history of previous hospitalization, previous antibiotic treatment, admission to intensive care units, advanced age, location at the time of infection and genetic characteristics 6,7.

Advances in molecular typing techniques, including pulsed-field gel electrophoresis (PFGE) 9, staphylococcal cassette chromosome mec (SCC*mec*) 10, Spa typing 11,12 and multilocus sequence typing (MLST) 13 have facilitated the study of clonal distributions of MRSA strains isolated in different countries and revealed a diversity in the genetic backgrounds of MRSA isolated in different geographical locations 14. In addition to the capacity to acquire antibiotic-resistance determinants, some MRSA strains have also acquired the ability to spread rapidly between patients within and between hospitals, thereby causing major problems for infection control. Hence, some epidemic MRSA strains have spread internationally 14. For example, the epidemic MRSA clones ST239-MRSA-III, ST22-MRSA-IV and ST30-MRSA-IV are widely distributed globally 8 whereas the USA300 MRSA is the dominant MRSA clone in North America and another MRSA clone, the ST80-MRSA-IV clone, is distributed widely in European countries, North Africa, the Middle East and the Gulf Cooperation Council (GCC) countries 15.

Studies on the distribution of MRSA clones in the GCC countries are limited 15–18. Although MRSA has been reported in the Sultanate of Oman since 1995 19, there are no data on the MRSA genotypes prevalent in the country. This study was conducted to determine the prevalence and distribution of MRSA clones in a tertiary hospital in the Sultanate of Oman.

## Materials and Methods

### Setting

The Sultan Qaboos University Hospital (SQUH) is a 550-bed teaching hospital of the Sultan Qaboos University. The hospital has 13 different medical departments, which include Surgery, Oral Health, Ophthalmology, Obstetrics & Gynaecology, Medicine, Human Clinical Anatomy, Haematology, Genetics, Family Medicine and Public Health, Emergency Medicine, Child Health, Behavioural Medicine, Anaesthesia and Intensive Care in addition to technical departments.

### MRSA isolates

A total of 79 non-repeat MRSA isolates obtained from clinical samples between March and December 2011 at the SQUH were investigated. Isolation and identification of MRSA from clinical samples were performed in the diagnostic microbiology laboratory of SQUH based on cultural characteristics, Gram stain, positive tube coagulase and DNAse tests. Methicillin resistance was confirmed by the amplification of *mecA* as described previously 20. The isolates were obtained from samples listed in Table1. Pure cultures of the isolates were preserved in Cryo-bank vials at −80°C. Molecular typing was performed at the Department of Microbiology, Health Science Centre, Kuwait University, Kuwait.

**Table 1:** Association of Panton–Valentine leucocidin-positive (PVL+) methicillin-resistant *Staphylococcus aureus* isolates with different types of infections

Types of infection	No. of strains	No. (%) of PVL+
Skin and soft tissue infections	29	21 (72.4)
Abscess	19	14 (73.7)
Ulcer^a^	4	2 (50)
Skin lesions/boils/furuncles	3	3 (100)
Folliculitis	1	1 (100)
Cellulitis	1	0 (0.0)
Blisters	1	1 (100)
Wounds	22	9 (40.9)
Postsurgical	11	3 (27.3)
Trauma	11	6 (54.5)
Respiratory tract infections	11	0
Pneumonia	4	0 (0.0)
Others^b^	7	0 (0.0)
Septicaemia/bacteraemia	6	3 (50)
Ear infection	5	1 (20)
Invasive infections (osteomyelitis/arthritis)	2	1 (50)
Colonization^c^	4	0 (0.0)
Total	79	35 (44.3)

a Bed Sore, pressure sore, mouth and groin ulcers and diabetic foot ulcer.

b Including cystic fibrosis.

c Nasal and umbilical.

### Antibacterial susceptibility testing

Antibacterial susceptibility testing was performed by the disc diffusion method 21 with the following antimicrobial disks (Oxoid, Basingstoke, UK): benzyl penicillin (2 U), cefoxitin (30 μg), kanamycin (30 μg), mupirocin (200 μg and 5 μg), gentamicin (10 μg), erythromycin (15 μg), clindamycin (2 μg), chloramphenicol (30 μg), tetracycline (10 μg), trimethoprim (2.5 μg), fusidic acid (10 μg), rifampicin (5 μg), ciprofloxacin (5 μg), teicoplanin (30 μg), vancomycin (30 μg) and linezolid (30 μg). Discs containing cadmium acetate (50 μg), propamidine isethionate (100 μg) and mercuric chloride (109 μg) were prepared in the laboratory. Minimum inhibitory concentration (MIC) for cefoxitin, vancomycin and teicoplanin were determined with E-test strips (AB BioMérieux, Marcy l'Etoile, France) according to the manufacturer's instructions. *Staphylococcus aureus* strain ATCC25923 was used as a quality control strain for susceptibility testing.

### SCC*mec typing*

SCC*mec* typing was performed by PCR assays as described previously 22,23.

### Detection of genes for Panton–Valentine leucocidin

All isolates were tested for the presence of *lukS-PV-lukF-PV*, which codes for Panton–Valentine leucocidin (PVL), in PCR assays using previously described primers and protocols 24,25. PCR products were analysed by agarose gel electrophoresis.

### Pulsed-field gel electrophoresis

The PFGE of *Sma*I-digested chromosomal DNA was performed as described previously 26. PFGE patterns were compared using Bioinformatics FPQuest software version 4.0 software (BioRad, Hercules, CA, USA) and Dice correlation coefficients, with optimization and band position tolerance set at 1.0% and 2.3%, respectively 27.

### Spa typing

Spa typing was performed as described by Harmsen *et al*. 12 for all MRSA isolates. DNA sequencing was performed using a 3130×1 genetic analyser (Applied Biosystems, Foster City, CA, USA) in accordance with the manufacturer's protocol. Isolates were assigned to particular Spa types using the Spa typing website (http://www.spaserver.ridom.de).

### Multilocus sequence typing

The MLST was performed on all isolates as described by Enright *et al*. 13. Isolates were assigned a sequence type (ST) according to the MLST website (http://www.mlst.net).

## Results

The 79 MRSA isolates were obtained from 46 male patients and 33 female patients. Forty-three patients were 19–59 years old, 26 patients were ≤18 years old and ten patients were ≥60 years.

Thirty-five (44.3%) MRSA isolates were positive for the presence of *lukS-PV-lukF-PV*, mostly in isolates that were associated with skin and soft tissue infections and septicaemia but not in isolates recovered from colonization or respiratory tract specimens (Table1).

### Antibiotic resistance of MRSA isolates

All 79 MRSA isolates were susceptible to vancomycin (MIC ≤ 2 mg/L), teicoplanin (MIC ≤ 2 mg/L), linezolid, tigecycline and mupirocin but were resistant to tetracycline (*n* = 24), erythromycin (*n* = 21), clindamycin (*n* = 19), kanamycin (*n* = 17), trimethoprim (*n* = 15), ciprofloxacin (*n* = 14) and fusidic acid (*n* = 12; 15.2%), gentamicin (*n* = 10) and streptomycin (*n* = 6). One isolate was resistant to chloramphenicol. Sixteen isolates expressed inducible resistance to clindamycin.

All 79 isolates were resistant to mercuric chloride and 68 (86.1%) isolates were resistant to ethidium bromide and cadmium acetate. Nine isolates were resistant to more than three classes of non-β-lactam antibiotics tested and were classified as multiresistant isolates.

### Molecular typing of MRSA isolates

**Figure 1:**
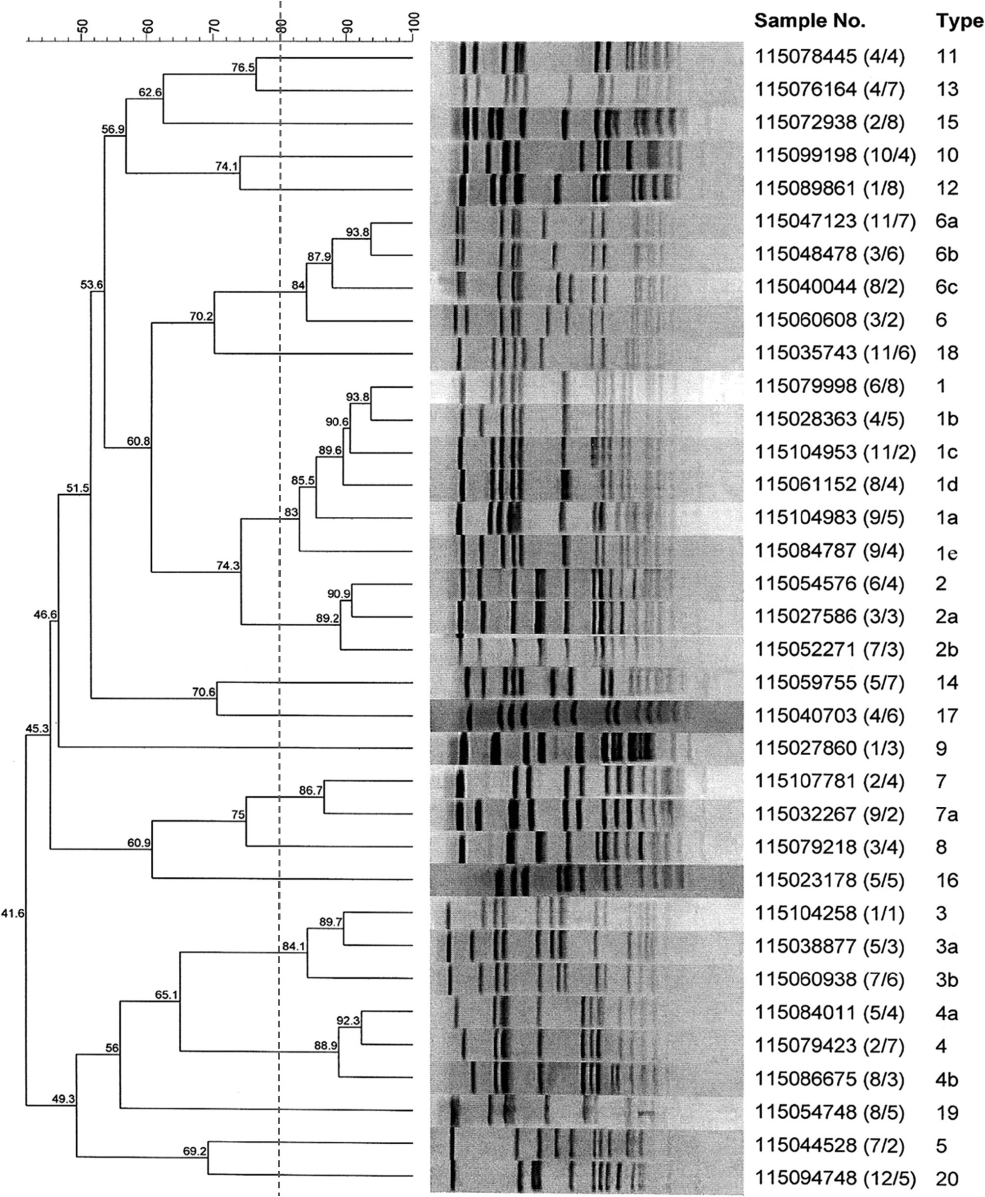
Dendrogram of pulsed-field gel electrophoresis patterns of methicillin-resistant *Staphylococcus aureus* isolates obtained from SQUH Oman.

Table2 summarizes the results typing the 79 MRSA isolates using PFGE, SCC*mec*, Spa typing and MLST. The isolates were classified into 19 PFGE patterns (Fig. 1), four SCC*mec* types, 25 Spa types and 21 sequence types.

**Table 2:** Characteristics of methicillin-resistant *Staphylococcus aureus* from SQUH

PFGE type	#	Antimicrobial-resistance pattern	SCC*mec*	Spa type	MLST	PVL
1	20	Cd, Hg, Eb	IV	t304	ST6	neg
	1	Hg	IV	t304	ST6	neg
	1	Cd, Hg	IV	t304	ST6	neg
	1	Cd, Hg, Em, Clin	IV	t304	ST6	neg
	1	Cd, Hg, Eb, Fd	IV	t304	ST6	neg
	1	Hg, Em, Clin	IV	t304	ST6	neg
	3	Hg, Eb	IV	t304	ST6	neg
	1	Cd, Hg	IV	t7966	ST6	neg
2	5	Cd, Hg, Eb, Tet	IV	t690	ST1295	pos (4)
	2	Cd, Hg, Eb, Tet, Em, Clin,	IV	t690	ST1295	pos (1)
	3	Cd, Hg, Eb, Tet, Cip	IV	t690	ST1295	pos (3)
	1	Cd, Hg, Eb	IV	t690	ST1295	pos
	1	Cd, Hg, Tet, Fd	IV	t690	ST1295	pos
3	1	Cd, Hg, Eb, Gm, Km, Sm, Tet, Fd	IV	t044	ST80	neg
	1	Cd, Hg, Eb, Km, Sm, Tet, Em, Clin, Fd	IV	t044	ST80	pos
	1	Cd,Hg, Eb, Km, Sm, Tet, Fd	IV	t044	ST80	pos
	1	Cd, Hg, Eb, Tet, Em, Clin	IV	t8154	ST450	neg
	1	Cd, Hg, Eb, Km, Em, Clin	IV	t304	ST6	pos
	1	Cd, Hg, Eb, Km, Sm, Tet, Fd	IV	t304	ST6	pos
4	4	Cd, Hg, Gm, Km, Em, Clin, Tp, Cip	V	t657	ST772	pos
	1	Cd, Hg, Eb, Gm, Km, Em, Clin, Tp, Cip	V	t657	ST772	pos
	1	Cd, Hg, Eb, Gm, Km, Em, Tp	V	t2085	ST573	pos
5	1	Hg, Em, Clin, Tp, Cip,	IV	t852	ST22	pos
	1	Hg, Eb, Em, Clin, Tp	IV	t852	ST22	pos
	1	Hg,Gm,Km,Em, Clin, Tp, Cip	IV	t852	ST22	pos
	1	Cd,Hg,Eb, Gm,Km, Tp, Cip	IV	t852	ST22	pos
6	1	Hg, Eb, Fd, Em, Clin	IV	t002	ST5	pos
	1	Cd, Hg, Eb	IV	t002	ST5	pos
	1	Hg, Eb, Km, Sm, Em, Clin, Fd	II	t003	ST5	neg
	1	Cd, Hg, Eb, Tet	IV	t855	ST628	pos
	1	Cd, Hg, Eb, Tet, Tp, Em, Clin, Cip	IV	t442	ST487	neg
7	1	Cd, Hg, Eb	IV	t021	ST30	neg
	3	Cd, Hg, Eb	IV	t019	ST30	pos
	1	Cd, Hg, Eb, Tet, Tp, Fd	IV	t019	ST30	pos
8	1	Cd, Hg, Tet	IV	t5686	ST188	neg
9	2	Cd, Hg, Eb, Km, Sm, Em, Clin, Cip	V	t315	ST361	neg
10	1	Cd, Hg, Eb, Tet, Cm	V	t688	ST627	neg
11	1	Cd,Hg, Eb	IV	t4447	ST631	neg
12	1	Cd, Hg, Eb, Em, Clin, Fd, Tp, Cip	III	t311	ST5	pos
13	1	Cd, Hg, Eb, Em, Clin, Fd	IV	t127	ST1	neg
14	1	Cd, Hg, Eb, Gm, Km, Em, Clin, Tet, Rf, Cip	III	t632	ST239	neg
15	1	Cd, Hg, Eb, Em, Clin, Tet, Tp, Cip	IV	t064	ST8	neg
16	1	Cd, Hg, Eb, Em, Clin, Fd	IV	t311	ST1197	pos
17	1	Cd, Hg, Eb, Km, Em, Clin, Cip	IV	t150	ST585	pos
18	1	Cd, Hg, Eb, Tet, Em, Clin	IV	t8213	ST63	pos
19	1	Hg, Eb, Tp	IV	t401	ST1802	neg

Antimicrobials abbreviations:

Cd, cadmium acetete; Clin, clindamycin; Cip, ciprofloxacin; Cm, chloramphenicol; Eb, ethidium bromide; Fd, fusidic acid; Gm, gentaimicn; Hg, mecuric chloride; Km, kanamycin; Rf, rifampicin; Sm, streptomycin; Tet, tetracycline; Tp, trimethoprim; MLST, multilocus sequence typing; PFGE, pulsed-field gel electrophoresis; PVL, Panton -Valentine Leucocidin; SCCmec, staphylococcal chromosome cassette mec.

Sixty-eight (86.0%) of the 79 isolates carried the SCC*mec*-IV genetic element while eight isolates (10.1%) carried SCC*mec*-V. Two isolates carried SCC*mec*-III and only one isolate carried SCC*mec*-II. SCC*mec*-I was not detected. A combination of the typing results revealed that ST6-IV/t304 (*n* = 30) was the most common genotype followed by ST1295-IV/t690 (*n* = 12), ST772-V/t657 (*n* = 5), ST22-IV/t852 (*n* = 4), ST30-IV/t019 (*n* = 4), ST80-IV/t044 (*n* = 3), ST5-IV/t002 (*n* = 2) and 15 sporadic genotypes. Based on these results, seven (8.8%) isolates consisting of four ST22-IV/t852, one ST239-III/t632, one ST5-III/t311 and one ST5-II/t003 were recognized healthcare-associated MRSA and 72 (91.2%) isolates that carried SCC*mec*-IV (*n* = 67) or SCC*mec*-V (*n* = 5) genetic elements were community-acquired MRSA.

## Discussion

This study has provided initial data on the prevalence and distribution of MRSA genotypes in the SQUH, a major tertiary hospital in the Sultanate of Oman. The MRSA isolates belonged to diverse genetic backgrounds with ST6-IV/t304 clone, detected in 39.2% of the isolates, as the dominant clone. The dominance of the ST6-IV/t304 clone at the SQUH in Oman was different from the situation in Saudi Arabia 15 and Qatar 18 where ST239-III-MRSA and ST30-IV-MRSA were the dominant MRSA clones, respectively. However, before this report, two ST6-IV/t304 strains were isolated at Tawam Hospital in the United Arab Emirates (UAE) in 2008 16. Interestingly, 28 of our ST6-IV/t304 isolates lacked genes for PVL and were susceptible to non-β-lactam antibiotics similar to characteristics of the strains from the UAE hospital 16. These observations may indicate the expansion of this clone in the GCC countries.

The other common MRSA clones detected in this study, ST30-IV/t019/t021, ST80-IV/t044, ST772-V/t657, ST5-IV/t002 and ST22-IV/t852 have also been reported previously in other GCC countries 15–18. However, although ST22-IV-MRSA has been reported previously in Saudi Arabia 15, Kuwait 7,17, UAE 16 and Qatar 18, the PVL-positive ST22-IV/t852 clones reported here share similarities with ST22-IV/t852 reported recently in Qatar 18 but differ from the ST22-IV/t005 isolated in the UAE 16. Furthermore, three of our four ST22-IV/t852 isolates were multidrug-resistant whereas ST22-IV, reported previously from Kuwait 17 and UAE 16, were PVL-negative and non-multiresistant. Therefore the ST22-IV/t852 strains may represent an emerging multiresistant variant of ST22-IV MRSA.

The results also showed that only 8.8% of the isolates—belonging to ST239-III, ST5-II, ST5-III and ST22-IV clones—were healthcare-associated MRSA. Therefore, 91.2% of the isolates carrying SCC*mec*-IV/V genetic elements were community-acquired MRSA. These reports highlight differences in the prevalence of MRSA clones in the GCC countries, strengthening the need for national surveillance for the clonal distribution of antibiotic-resistant pathogens in these countries.

The study also revealed that 44.3% of the isolates carried genes for PVL. This was higher than the 14.6% PVL gene-positive MRSA reported recently in a Kuwait hospital 28 but lower than the 54.2% positive rate obtained in a Saudi Arabian hospital 15, indicating the diversity of MRSA bearing PVL genes in the GCC countries. PVL gene-positive *S. aureus* have been associated with necrotic skin lesions and community-acquired necrotic pneumonia 24. In this study PVL gene-positive MRSA were obtained from skin and soft tissue infections but not from respiratory tract infections. However, the significance of this observation is uncertain because of the small number of MRSA isolated from respiratory tract.

In conclusion, this study has presented the first data on the distribution of MRSA genotypes at the SQUH in Oman. The MRSA isolates belonged to diverse genetic backgrounds with a predominance of CA-MRSA clones comprising ST6-IV/t304 and ST1295-IV/t690, followed by ST772-V/t657, ST30-IV/t019/t021 and ST80-IV/t044.
